# Are Patents Impeding Medical Care and Innovation?

**DOI:** 10.1371/journal.pmed.1000208

**Published:** 2010-01-05

**Authors:** E. Richard Gold, Warren Kaplan, James Orbinski, Sarah Harland-Logan, Sevil N-Marandi

**Affiliations:** 1 Faculty of Law, McGill University, Montreal, Quebec, Canada; 2 Department of International Health, The Boston University School of Public Health, Boston, Massachusetts, United States of America; 3 St. Michael's Hospital, University of Toronto, Toronto, Canada; 4 Harvard University, Cambridge, Massachusetts, United States of America; 5 University of Toronto, Toronto, Canada

## Abstract

*Background to the debate:* Pharmaceutical and medical device manufacturers argue that the current patent system is crucial for stimulating research and development (R&D), leading to new products that improve medical care. The financial return on their investments that is afforded by patent protection, they claim, is an incentive toward innovation and reinvestment into further R&D. But this view has been challenged in recent years. Many commentators argue that patents are stifling biomedical research, for example by preventing researchers from accessing patented materials or methods they need for their studies. Patents have also been blamed for impeding medical care by raising prices of essential medicines, such as antiretroviral drugs, in poor countries. This debate examines whether and how patents are impeding health care and innovation.

## Abstract 2

This month's debate examines whether the current patent system is crucial for stimulating health research or whether it is stifling biomedical research and impeding medical care.

## E. Richard Gold's Viewpoint: We Could Increase the Productivity of Biomedical Innovation Systems by Rethinking How We Use Patents

The question posed in the title of this *PLoS Medicine* debate seems to be a simple one, but there is a complex spectrum of answers depending on how one interprets the question. In this article, I lay out four different interpretations and their corresponding answers.

### How Are Existing Patent Rights Impeding Medical Care and Innovation?

The narrowest version of the question focuses on the effect of existing patents held by actors (industry, university, government laboratories, etc.) on medical care and innovation.

In high-income countries, the evidence suggests that existing patents increase the cost of medicines [1]. Whether patents increase the cost of other services, such as diagnostics, is unclear [2]. For example, in their recent analysis of patents on genetic testing, Robert Cook-Deegan and colleagues concluded that “prices of patented and exclusively licensed tests are not dramatically or consistently higher than those of tests without a monopoly” [2]. What impact do existing patents have on the total cost of medical care in rich countries? Again, the evidence is unclear. Patents could conceivably reduce the total cost of care if new patented medicines turn out to be cheaper than existing medical interventions.

In those low- and middle-income countries in which current medications are subject to patent rights, existing patents seem to make medicines more expensive and increase the difficulty of creating novel mechanisms through which to deliver medicines [3],[4]. In all countries, existing patents make research and development more expensive for the simple reason that researchers and companies must clear patent rights to do their work. Whether this cost is offset by other benefits is a subject I turn to next.

### How Is The Prospect of Obtaining Patent Rights Impeding Medical Care and Innovation?

The theory underlying patent rights is that patents encourage people to invest in bringing a compound through clinical trials and into practice [5],[6]. The prospect of future patents may, therefore, increase innovation today and may increase medical care by encouraging manufacturers to introduce new medicines [7]. While pharmaceutical companies spend almost twice as much on marketing than on research [8], they nevertheless invest heavily in developing new medicines.

Two questions remain, however. First, while patents provide an incentive to bring a new product to market, are these incentives better than those provided by alternative mechanisms? We know that existing business strategies of both pharmaceutical and biotechnology companies rely heavily on patents [6],[9], but this does not prove that they could not have developed strategies that did not rely on patents. It appears that the biomedical industry's reliance on patents is historically arbitrary [10], rather than being necessary to spur innovation. So, for example, would a prize awarded to those who discover new medicines be a better mechanism than using patents [11]? Neither theory nor evidence provides a clear answer. Second, are the benefits of patents in encouraging the development of new medicines offset by the increased prices we pay for existing medicines and by the higher fees that researchers must pay? Again, empirical research is inconclusive but is strongest in the biomedical sector [10]. In the end, we have no better answer today than in the 1950s when economists Edith Penrose and Fritz Machlup concluded that the evidence supporting or undermining the patent system is lacking [12],[13].

### How Is The Patent System Impeding Medical Care and Innovation?

If we look at the outcomes of biomedical innovation, a different answer emerges. The patent system—not just patent rights but how they are obtained and used—has resulted in an innovation system characterized by a dramatic increase in health care costs and decreasing (quantitatively and qualitatively) levels of innovation, especially by dollar spent [9]. While one cannot say that these problems are inherent in patent law they are, nevertheless, an outcome of the manner in which actors deploy patent rights.

The evidence points to a crisis in biomedical innovation even if not to a solution. While health care costs are increasing rapidly, the fastest growing component of those costs are pharmaceutical products [14]. The costs of developing a new medicine from discovery through clinical trials appear to double every decade [15]. Yet, despite increasing investments in research and development, industry is producing fewer new drugs every year of which a declining percentage is truly innovative [16]. Beyond this, investments in the health needs of developing countries remains very low by any standard, and patents continue to get in the way of modifying existing medicines for the needs of those countries [3].

All of this shows an industry in serious difficulty and a health care system facing unsustainable cost increases and fewer new products. There are many reasons for this crisis that stretch well beyond the patent system. To the extent, however, that the industry's current business models are build around patents, the patent system itself must shoulder its share of responsibility.

### Would a Different Deployment of Patents Impede Health Care and Innovation?

While some nongovernmental organizations call for the creation of alternatives to the patent system, such as prize systems [11], these are even less proven than the patent system in sustaining innovation and increasing health care. A more practical solution to the problems described above is not to do away with patent rights but to use them more wisely. This means everything from deciding not to apply for patents over certain inventions, as does the Structural Genomics Consortium [17], to licensing out patent rights widely for research purposes, as does the Health Commons (http://www.healthcommons.net/), through to exploring the possibility of creating “open source” licenses in biomedicine [18]. None of these strategies require changing patent laws, but all present a serious challenge to the way that universities and industry obtain and deploy patent rights.

### Conclusion

There are many questions buried within the question “*How are patents impeding medical care and innovation?*” At bottom, we do know that our biomedical innovation system is too expensive and too unproductive and that patents play an important role within that system. If, in the end, it is results that count, then it is certainly time to question the business models that sit on top of our existing patent system [9],[18].

## Warren Kaplan's Viewpoint: The Evidence on Whether Patents Impede Medical Innovation Is Ambiguous

The complicated debate about whether or not patents impede “downstream” medical care and “upstream” medical innovation is ultimately about *access* to such care and innovation, which are at opposite ends of a “chain” of biomedicine.

### Access to Medical Care

Clinical research is costly, lengthy, and high-risk. Pharmaceutical companies apply for patents on new drugs to gain market exclusivity for a limited period. The aim of this exclusivity is to generate revenue from sales and recoup the substantial cost of drug development by collecting fees (“royalties”) from users of the patented technology. Pharmaceutical companies also fund their research from these fees. Patented pharmaceuticals cost more than identical medicines that are off-patent. Access to medicines is inhibited by high prices. Patents are a factor in inhibiting access to pharmaceutical treatment, particularly in low- and middle-income countries [8],[20]–[22].

For medical devices, large manufacturers continue to benefit from price increases on patent-protected devices. As with pharmaceuticals, iterative improvements in performance and safety of existing devices are typically patented. These patents can, in principle, create barriers to market entry for new competitors. Nonetheless, the literature on the impact of patents on access to medical devices is slim compared to that for pharmaceuticals.

The impact of gene patents on genetic testing has garnered much recent press and academic interest [23]–[26], including a lawsuit by the American Civil Liberties Union against Myriad Genetics charging that patents on two human genes associated with breast and ovarian cancer stifle research that could lead to cures [27]. The lawsuit argues that the patents on these genes are unconstitutional and invalid. But in fact, there is little in the way of consistent evidence to suggest that gene patents inhibit patient access to diagnostic tests, at least in the United States [24]. Even so, when Mildred Cho and colleagues interviewed 132 directors of clinical genetic testing laboratories, 53% of respondents reported that patents or licenses *had* impeded their ability to develop and provide genetic tests [28].

Patents are a critical factor affecting access to medical care, but they are not the only factor. Other factors influencing medical care include demand for a product and market size (e.g., a large market and high demand for a product might lead to considerable revenue for the company even at a lower price),

### Access to Innovation

The proliferation of patents may block biomedical R&D because researchers are unable to obtain the many different permissions required (e.g., permission may be required to use patented reagents, to try a patented method, and/or use a patented device) [28]. This situation has been called the “anticommons” problem [29]—R&D is inhibited by the presence of many intellectual property owners' exclusive and possibly conflicting rights over devices and methods needed to perform R&D on biomedical products.

However, there is little empirical evidence that an anticommons problem is impeding innovation. For example, the French Community Innovation Survey found that 14% of R&D collaborating firms had to abandon or delay their innovation projects because of difficulties in their partnerships (“cooperation failures”), and the survey explored reasons for such failures. Intellectual property rights were *not* a cause of cooperation failure—in fact, the authors found that “industries where firms are able to better appropriate their research results (through patents, models and secrecy) present lower rates of ‘cooperation failures’” [30].

In their analysis of how patents affect medical innovation in Australia, Dianne Nicol and Jane Nielsen concluded that “in general the Australian industry seems to be avoiding an anticommons situation, but the potential still exists for its emergence” [31]. In the US and Japan, there is also very little evidence of an anticommons problem preventing innovation [32]. John Walsh and colleagues surveyed 507 academic biomedical researchers, asking them about the impact of patents on access to the knowledge and material inputs that are used in subsequent research [33]. The authors concluded that “access to knowledge inputs is largely unaffected by patents.” A survey of 70 attorneys, scientists, and managers in the biomedical research industry did not find evidence of the anticommons problem [34].

In contrast, Stephen Meurer has shown that the anticommons problem prevented a group of about 100 academic biologists from building a worldwide human mutations database [35]. The biologists tried to trade their data for corporate support of the database. Although they received an offer of US$2.3 million, a deadlock occurred because most members of the group could not afford the information costs needed to reach a decision—a prediction of the anticommons.

Access to materials and/or data—such as cell lines, reagents, genetically modified animals, and unpublished information—can be restricted if these are owned by other researchers. In a survey of agricultural biologists [36], and based on my own experience in biotechnology, delayed or blocked access to such materials results from having to negotiate material transfer agreements (the University of California, Berkeley defines a material transfer agreement as “a contract that governs the transfer of tangible research materials between two organizations, when the recipient intends to use it for his or her own research purposes” [37]). Restrictions on access do not appear to depend on whether the material is itself patented [38]. Typically, no issued patents exist on such materials covered by these material transfer agreements. But it is the *possibility* of future patent protection and the desire on the part of the supplier to manage this uncertainty that slows down or even eliminates such transfers of technology.

### Conclusion

What are we to make of all this? The actual evidence on whether patents impede innovation or inventiveness in biomedicine is, in a word, ambiguous. Yet firms clearly tend to avoid research projects for which there are many existing patents [39]. Both the process of determining which potentially relevant patents are important to a research project and the negotiations for access to them can delay, but less often kills, innovation. In industry and universities, researchers adopt strategies of “licensing, inventing around patents, going offshore, the development and use of public databases and research tools, court challenges and … using the technology without a license (i.e. infringement) to achieve their particular goals” [39].

This raises the question, What are these various “design around” actions manifestations of, if not actual patent blockages or threats of the same? We act as if the anticommons block to innovation is real. Perception is reality. Patents, or perhaps only the fear of their enforcement, inhibit biomedical innovation. If we knew how strong the inhibition really was, we would be having a different debate.

## James Orbinski's, Sarah Harland Logan's, and Sevil N-Marandi's Viewpoint: Patents Skew Biomedical Research Toward Problems of the Rich World

If patents represent a bargain between the claimant to intellectual property (IP) and the state, and on balance should benefit society, a key question in this age of globalization is “which society?” The United Kingdom's Royal Society, an independent academy of science, rightly argues that “uses of intellectual property that benefit people in one part of the world but conspicuously fail to benefit others, or even act to their detriment, are not what the [patent] system is supposed to be about” [40].

For developing countries, patents can impede medical care by pricing medicines and other health care technologies (HCTs) out of the reach of patients or their health care systems. Pharmaceutical companies have little interest in pricing drugs for developing country markets because they are seeking to maximize global not national profits, and do not want to set a low price precedent that would increase demand in wealthy countries for similar low prices [41]. For those with a purchasing power less than what is needed to meet minimal needs—i.e., most of the 3.8 billion people who live on less than US$2 per day [42]—access to HCTs is little more than a discomforting dream. Further, if a treatment is too expensive, other factors that can affect medicines availability, such as drug distribution systems and rational drug use policies, become moot. Indeed, it was only when generic competition lowered the price of antiretroviral therapy for HIV—from more than US$15,000 per patient per year in 2001 to less than US$99 in 2007—that the policy debate shifted from whether such therapy was possible in resource-poor settings to how to strengthen health infrastructure to provide comprehensive HIV health care for people in such settings [43],[44].

To increase access to existing HCTs, governments can make use of fully legal safety provisions of the World Trade Organization's Trade in Intellectual Property Rights Agreement (TRIPS). These provisions include compulsory licensing, which allows a government to force a drug company to license its patent to a local generic producer who must pay a royalty to the patent holder. But a government is allowed to issue a compulsory license only after price negotiations with the patent holder have failed. Nevertheless, compulsory licensing remains a valuable tool, as memorably shown in 2001 when South Africa issued compulsory licenses to produce selected anttiretroviral drugs. Although 39 pharmaceutical companies attempted to sue South Africa's government for allegedly infringing on their patent rights, they ultimately chose to withdraw this lawsuit in the face of immense public pressure [45]. The confrontation led the World Trade Organization to issue its November 2001 Doha Declaration, which affirmed that “the TRIPS Agreement does not and should not prevent members from taking measures to protect public health” [46].

Current patent laws also skew biomedical research to products that yield high profits rather than to global priority health needs in both developed and developing countries. Currently, malaria, pneumonia, diarrhea, and tuberculosis, which together account for 21% of the global disease burden, receive 0.31% of all public and private funds devoted to heath research [47],[48]. More than 1 billion people—the overwhelming majority of whom are in the developing world—suffer from neglected tropical diseases, those for which there are inadequate or nonexistent treatments and a paucity of research and development [49]. Of the 1,556 new pharmaceutical compounds that appeared on the market between 1975 and 2004, just *twenty* of these drugs—1.3%—were for tropical diseases and tuberculosis [50].

The international debate around patents has been largely framed in terms of “protection for” versus “access to” IP. If the framing of the debate shifts to a focus on research and development, this is likely to strengthen the leverage of developing countries to change the dynamics of IP negotiations in trade agreements [51]. Entirely shifting the debate from IP rights to the R&D gap may help tackle the fundamental problem of a monopoly-based innovation and access system. One example is nonexclusive licensing practices, such as those used by the not-for-profit Drugs for Neglected Diseases Initiative (http://www.dndi.org/). The initiative finances R&D up front and offers the outcome of its research on a nonexclusive basis to generic producers, allowing for technology transfer and competition among multiple producers [51]. Furthermore, universities currently hold important patents on many life-saving drugs, including the antiretroviral drugs stavudine (Yale University), abacavir (University of Minnesota), lamivudine (Emory University), and enfuvirtide (Duke University) [52]. In recognition of these university patents, Universities Allied for Essential Medicines (http://www.essentialmedicine.org) proposes that “when a university licenses a promising new drug candidate to a pharmaceutical company, it should require that the company allow the drug to be made available in poor countries at the lowest possible cost” [53]. Another alternative to overcoming current patent barriers is the use of patent pools, as proposed by the WHO, Médecins Sans Frontières, and UNITAID [54],[55]. Here, a number of patents held by different entities, such as companies, universities, or research institutes, are pooled and made available to others for production or further development—of, for example, pediatric formulations or fixed-dose formulations. The patent holders receive royalties that are paid by those who use the patents. The pool manages the licenses, the negotiations with patent holders, and the receipt and payment of royalties.

Other innovative policy proposals, such as the Heath Impact Fund (a strategy to create a publicly funded “pot of gold” that would attract the private sector to create R&D innovations that effectively address priority global heath needs) [56], should be implemented. However, using patents as the financial incentive to encourage the pharmaceutical industry to develop drugs for the world's poor is of limited use where the market is nonexistent because neither governments nor patients can afford the end product [57]. Instead, framing the issue around global R&D, as opposed to international IP rights, will aid in developing public–private partnerships and a set of novel policy alternatives that support approaches to addressing the public health needs of developing nations [58].

The patent system as it affects access to and innovation for HCTs is broken. The system must be reformed so that public goods—such as genuine innovation and access to HCTs—are not sacrificed on the altar of private gain. This reform must prioritize the public good, use innovative policy tools to harness the private sector where it is possible to do so, and create public R&D capacity where market forces and actors are likely to continue to fail.

## Abbreviations

HCT: health care technology
IP: intellectual property
R&D: research and development
TRIPS: Trade in Intellectual Property Rights Agreement
